# Algorithmic Decision-Making, Delegation and the Modern Machinery of Government

**DOI:** 10.1093/ojls/gqaf018

**Published:** 2025-06-10

**Authors:** Oliver Butler

**Keywords:** algorithmic decision-making, automated decision-making, delegation, ADM

## Abstract

The development of the principle of non-delegation in administrative law was a response to the perceived needs of a ‘modern machinery of government’, which emerged in post-war 1940s Britain. While it ostensibly sought to ensure that decision-makers appropriately retain their decision-making discretion, and through that political accountability, it has developed into a permissive doctrine that facilitates significant delegation of decision-making within public administration. As algorithmic decision-making (ADM) is increasingly used in public decision-making, it is necessary to question whether it remains fit for the modern machinery of government of the 2020s and beyond. This article considers the limitations of the doctrine in the context of public ADM, considers the shift in doctrinal approach that would be needed to accommodate this emerging machinery and concludes that the doctrine faces serious challenges in accommodating ADM in public decision-making.

## 1. Delegation and the ‘Modern Machinery of Government’

In administrative law, the principle of non-delegation seeks to ensure that the decision-maker to whom a public function has been allocated does not unlawfully delegate that function to another. It reflects key constitutional principles: respect for the sovereign Parliament’s allocation of functions, the retention of responsibility for decision-making and therefore accountability for its exercise and transparency about its location. With political accountability to Parliament and legal accountability to the courts, through conventions of ministerial responsibility and duties of candour respectively, it is a core value of legitimate public decision-making.^2^ At first sight, therefore, it might be thought to be a valuable tool with which to maintain an appropriate degree of responsibility and accountability for, and transparency in relation to, complex decision-making structures in public administration, including the growing use of algorithmic decision-making (ADM).

The principle of non-delegation is not absolute and the courts have long recognised that there will be circumstances in which public functions can be appropriately delegated. The law is realistic about the necessity of delegation and its desirability in many contexts. The most significant exception to the principle of non-delegation is the *Carltona* principle,^3^ which recognises the extensive role of departmental civil servants as the *alter ego* of a minister,^4^ able to make decisions in the name of that minister. The *Carltona* principle has expanded considerably since the 1940s, to extend to arm’s-length departmental agencies.^5^ Similar permissive exceptions to the principle of non-delegation apply to other public functions.^6^

The advantages and disadvantages of the law on delegation in the context of traditional public decision-making are well known.^7^ The development of the *Carltona* principle was driven by a recognition of the practicalities and constitutional realities of the ‘modern machinery of government’, as it had developed by the 1940s.^8^ That ‘modern machinery’ was the expansion of traditional ministerial departments and the delegation of decision-making within a hierarchical bureaucratic structure. This was a machinery that required civil servants to make thousands of daily, individual decisions within an expanding welfare state.

It would be misleading to call this the ‘modern’ machinery of government today. While extensive decision-making still takes place through traditional means, there is increasing reliance on ADM within government, and in particular machine-learning algorithms to help make recommendations and classifications.^9^ In the UK, the Public Law Project (PLP) has sought to identify governmental uses of ADM in its Tracking Automated Government (TAG) project. The TAG published a register of its findings in 2023, containing details on 55 decision-making tools in use by UK public authorities. The PLP has expressed concern about transparency and accountability in the development of such tools.^10^

These technologies can be procured by government departments and public authorities from private developers, and the technical expertise required to develop them will mean that they are not often developed internally. The extent to which such technologies are procured ‘off-the-shelf’ or are subject to ‘bespoke’ requirements is unclear. Even where public authorities co-produce a bespoke algorithmic tool, important design choices could be delegated to developers. Automation bias (the unconscious bias in favour of a recommendation or classification of an automated process), constraints of time to review algorithmic outputs, a lack of understanding of the limitations of an algorithmic output and a lack of alternative evidence with which to challenge such an output could all in practice severely limit the ability of decision makers to retain their own decision-making discretion. The autonomy of public decision-makers risks being undermined by such delegation, and with it responsibility, accountability and transparency in public decision-making.

The law on delegation, as it currently stands, has a variety of limitations when addressing this new machinery of government, which could perpetuate, if not exacerbate, problems with accountability for public ADM. This article examines those limitations, considers the shift in doctrinal approach that would be required to accommodate this emerging machinery and concludes that the doctrine of non-delegation faces serious challenges in doing so. Even if adapting the principle of non-delegation to public ADM could go some way to addressing accountability, responsibility and transparency concerns, it is, of course, not a panacea, and would need to take place alongside a wider set of reforms.

There are a number of other approaches which might help to appropriately retain responsibility, accountability and transparency in the design, development and deployment of public ADM. While it is not the intention of this article to evaluate them, an important implication of the limitations of non-delegation identified in this article is that more attention must be given to evaluating those alternatives in the future. Non-delegation faces serious challenges in ensuring that discretion is appropriately retained by official decision-makers. Legislation might be required to identify important design choices in emerging public ADM systems and to subject them to appropriate retention by decision-makers. Algorithmic risk assessment and transparency requirements, such as the UK government’s Algorithmic Transparency Recording Standard,^11^ may need to be developed and expanded to help hold decision-makers accountable for delegated design choices. Public procurement processes might need reform,^12^ greater supply chain transparency sought^13^ and in-house expertise developed which is capable of scrutinising choices made by private developers. The limitations of administrative law in this regard also underline the importance of the development of the concept of ‘responsible design’ as part of the professional and ethical commitments of developers. This operates in parallel to legal responses and might facilitate customisation by public decision-makers and understanding of the trade-offs they entail, thereby empowering officials to retain their discretion appropriately.^14^

## 2. Public Algorithmic Decision-Making

Algorithmic decision-making refers to ‘the use of algorithmically generated knowledge systems to execute or inform decisions’.^15^ Importantly, therefore, it can take the form of autonomous decision-making systems, in which a decision is generated by an algorithm without any further human intervention beyond the development and deployment of the system, or augmented decision-making systems, in which human decision-making is supported by an algorithmic output. Sometimes referred to as decision-support tools, augmented decision-making systems commonly provide recommendations or initial classifications, which a human decision-maker reviews and acts upon.

This article includes both automated and augmented decision-making within its definition of algorithmic decision-making, and uses the abbreviation ‘ADM’ in this inclusive sense throughout. ADM can be used to refer more narrowly to fully automated decision-making.^16^ It is important in this article, however, to adopt the broader understanding of ADM for two reasons. First, even ‘fully’ automated decision-making has humans ‘in the loop’, even if that human involvement is at the development stage or the moment of decision to use automated decision-making for a set of decisions. Decisions relating to development and application are important sites of decision-making, and the human responsibility for those decisions should not be obscured. Secondly, modern government uses a broad range of automated and augmented decision-making technologies, interweaving human and machine decision-making, and a broader definition of ADM better captures the full range of relevant activity. It is reliance on machine learning that unites both automated and augmented decision-making technologies, and that technology forms the focus of this article.

Several high-profile controversies in the UK have drawn attention to the capacity of inadequate ADM systems to cause serious harm. In 2014, following a BBC Panorama investigation into exam fraud in the Test of English for International Communication, an English-language test used in applications for student visas, the Home Office required the exam provider ETS to identify cases of cheating using proxy candidates.^17^ ETS used a voice-recognition algorithm to flag suspected cases of cheating in its test recordings. Despite flagging (an implausibly high) 97% of 58,000 test results as fraudulent, the results were then relied upon by the Home Office to withdraw thousands of student visas. Legal challenges are ongoing and the Home Office’s approach to the scandal has been criticised by the Public Accounts Committee.^18^

In 2018, the Metropolitan Police’s Gangs Violence Matrix was subject to enforcement notices by the Information Commissioner’s Office for multiple breaches of data protection principles.^19^ It was a risk-scoring algorithm to classify potential offenders and victims of gang violence, based on various sources of intelligence, including social media posts and connections, and was discontinued in October 2022.^20^ Amnesty International UK has criticised the Gangs Violence Matrix for its disproportionate representation of young, black men.^21^

In 2020, Ofqual used an exam results algorithm to moderate centre-assessed grades awarded in lieu of examinations, which had been cancelled as part of the UK’s lockdown in response to the COVID-19 pandemic. The algorithm downgraded 39% of centre-assessed grades based on historic school performance. It was criticised for its unfairness and indirect socioeconomic discrimination, as centre-assessed grades from schools and courses with small class sizes were not moderated, and this correlated strongly with fee-paying schools. The moderated results were reversed following public outcry.^22^

Such ADM is the subject of increasing interest and concern in data protection,^23^ privacy,^24^ anti-discrimination^25^ and employment law.^26^ The regulation of artificial intelligence systems is now the subject of the European Union’s AI Act, which received final approval in May 2024.^27^ The UK government uses the Algorithmic Transparency Recording Standard to provide information about its use of algorithmic tools, although it does not currently extend to the wider public sector and is based on guidance, rather than being a statutory obligation.^28^ Although the UK does have guidelines for AI procurement for public authorities,^29^ the ability of UK procurement law to adequately regulate the development of AI for public sector uses have been questioned.^30^

There are many concerns about the impact of these technologies on important values, such as transparency, accountability, responsibility, inclusion, and privacy, and the potential of them to disrupt, for better or for worse, current approaches to law, regulation and governance. It would not be possible to do justice to all facets of those concerns in one article. This article engages with the increasing body of work that draws attention to the potential role of administrative law in regulating these technologies when they are used for public decision-making.^31^

The particular problem with public ADM that the article seeks to address is the concern that it obscures responsibility, accountability and transparency for the important design choices that embed value judgments and trade-offs in the development and implementation of the technology. Those are matters that should properly be retained by appropriate public decision-makers and not left to developers. Cobbe has called for a closer examination of administrative law doctrines in light of automated decision-making, including a call for a closer examination of the law on non-delegation.^32^ This article is an answer to that call. Casale has argued that limits on delegation could ‘offer significant protections’ in the context of the public use of machine-learning algorithms.^33^ This article argues instead that the current law has more significant limitations in that regard.

Writing extrajudicially, Lord Sales has expressed concerns about the impact of ADM on both individual and collective human agency, noting the need to find ‘ways of trying to close the gap between democratic, public control and technical expertise’.^34^ He has identified, among other legal doctrines, non-delegation as an area that ‘may assist in delineating the line between using technology to discharge a statutory function, and abdicating responsibility to that technology’.^35^ Most recently, he expressed the view that the legal approach which applies to delegation within a department or to a human decision-maker cannot be assumed to equally apply to ‘“delegation” to an automated system’.^36^ There is some reason to think, then, that there is a judicial appetite to reappraise traditional thinking around non-delegation in administrative law. This article seeks to contribute to legal thought on the viability of non-delegation doctrine as a response to ADM and the challenges it faces.

ADM systems commonly make use of structured machine learning. Machine learning describes a set of algorithmic techniques that seek to predict values or to classify objects based on correlations identified in a training dataset, and usually verified against a testing dataset, before being applied to new data. Such algorithms create complex statistical models, which are then applied as decision-making tools. We must be careful not to assume a simple mathematical rationality or neutrality in such tools. Important choices are necessary in the design and deployment of algorithms: the choice of machine-learning technique; the choice of training and testing datasets; and the choice between algorithmic models (developed by different techniques or on different datasets) to be applied to a new or changing context. Of course, there is no easy line between design and deployment. As with any socio-technical system, the division between design and implementation is blurred. This is sometimes because those who design a system oversee its implementation. Sometimes, it might be because feedback loops result in the original design parameters being updated. Sometimes, it is because discretion at the implementation stage is an intentional feature of a system or simply unavoidable, where algorithmic outputs must still be interpreted and applied. Complex socio-technical systems are commonly co-produced by many parties in this manner. The importance of thinking about design choices here is to emphasise that important decisions are made across the process of developing and implementing ADM and not simply when a ‘final’ decision is produced. This is a distinction of particular relevance in relation to non-delegation in administrative law, which places such an emphasis on the ‘final’ decision.

Different algorithms do not produce equally good solutions to the same problem. An algorithm trained on one dataset may perform better or worse than another when applied to a different dataset. Although the complexity and number of calculations used by machine-learning algorithms produces opacity as to the precise basis for their recommendations,^37^ there are various ways in which algorithms can be compared. Applying a trained algorithm to a testing dataset can reveal false-negative and false-positive rates against true-negative and true-positive rates, including against different segments of the database. This can reveal the bias of an algorithm and the extent to which different groups are disproportionately affected by that bias. A comparison of the resulting ‘confusion matrix’ for different algorithms trained on different datasets facilitates choice as to the trade-offs to be made between them,^38^ as do other ‘metrics and processes to detect discrimination’.^39^ Identifying the appropriate trade-off may involve difficult judgments. To the degree that the deployment context differs from the training and testing datasets, these measures may prove inaccurate. Measures of confidence in the testing results can vary depending on the new context in which an algorithm is deployed. Further important choices are therefore required as to the appropriateness of deploying an algorithm in a new context or to a different demographic.

These important choices can involve difficult trade-offs and exercises of discretion. Yeung and Lodge highlight the importance of ADM as a ‘design-based … modality of control’.^40^ Harkens has argued that ‘the design of … algorithmic tools involves a number of technical trade-offs and policy choices that can have a severe effect on the form of “fairness” which a given tool can provide’.^41^ Oswald similarly notes the risk of ‘decision-making being brought forward to the technical stages of a system’s design’ if appropriate steps are not taken.^42^ The Open Society Foundation emphasises that despite the characterisation of algorithmic scoring ‘as benign, neutral and objective’, it is ‘always constructed based on the goals, interests and cultures of institutions and individuals’, including ‘case workers, department heads, and the developers of algorithms’.^43^ This co-production of ADM can mask who is making those important choices about design and development, to the detriment of accountability and contestability. Jasanoff has argued that there is an ‘urgent need for participation mechanisms for engaging the public to play more active roles in the design and management of their technological futures’.^44^ Ensuring that important choices about algorithmic design and deployment are taken at a sufficient level of seniority may enhance the accountability and contestability of such technologies. There is good reason to think that this does not occur in practice. Veale and Brass explain that ‘ministers and civil servants do not specify the training parameters or data sources of algorithmic systems’ and ask ‘where are decisions for acceptable performance … made …?’.^45^ This leaves ‘significant discretionary, or at the very least agenda-setting, power to … “system level” bureaucrats’.^46^

Clearly, there is space for some level of appropriate reliance on the expertise of algorithm designers, and there are potential efficiency and consistency benefits to using ADM well. The problem of expertise in decision-making is well explored.^47^ In some respects, the problem of public ADM bears a similarity to reliance on expert advice more broadly. There is a risk of undue deference to the expert or algorithmic recommendation or classification. In part, this is because it is difficult to scrutinise the quality of a claim to expertise, and, in part, it is because expertise and algorithmic outputs legitimise consistent decisions and delegitimise divergent decisions, particularly if the decision-maker lacks an alternative evidence base for decision. A key difference between human expertise and algorithmic decision support, however, is that the latter is designed, and this might offer a more manageable number of decision points over which a responsible decision-maker could maintain discretion. While it might not be feasible for a senior decision-maker to review the many decisions taken by bureaucrats to whom public functions are delegated every day, it is far more feasible for such an individual to take the important design decisions that shape algorithmic outputs.

## 3. Limitations of the Principle of Non-delegation

Despite the plausibility of the principle of non-delegation to address inappropriate delegation to developers, there are three key features of the law on non-delegation and its exceptions that limit its ability to ensure that public decision-makers retain adequate discretion over important design choices in the co-production of ADM. These may perpetuate or even exacerbate the problem in the context of public ADM.

The first feature is a focus on the delegation of the ‘final decision’, as opposed to decision-making and exercises of discretion that lead to that ‘final decision’. The second feature is a tendency to analyse the permissibility of delegation as an ‘all or nothing’ matter, rather than to consider whether elements of discretion should be retained while others may be delegated. This is a tendency whose significance is amplified by the importance given to the ‘inevitability’ of delegation as an indicator that a power to delegate should be implied. The third is the scope of the *Carltona* principle itself. On the one hand, a permissive approach is taken to ministerial devolution of decision-making powers to a wide range of civil servants. Little scrutiny of decisions to delegate ‘internally’ is possible, due to that permissive approach and the application of mere rationality review to decisions to delegate, once it is so established that a power is delegable. On the other hand, there is serious doubt over the wider application of the *Carltona* principle to private actors involved in decision-making, which can either inhibit adoption of ADM or encourage reliance on a ‘final decision’ being made by an official. Together, these limitations make it difficult to apply principles of non-delegation to public ADM where important design decisions have been taken by private developers, rather than accountable decision-makers.

The unwillingness of the law on non-delegation to look to important choices made earlier in the decision-making process, which shape outcomes, its tendency to treat the permissibility of delegation as an ‘all or nothing’ matter, rather than separating out the most important aspects of decision-making for retention, and its permissiveness providing that a ‘final decision’ is taken by an official for whom the ultimate decision-maker is notionally accountable make the law ill-equipped to require the retention of important design choices by responsible senior decision-makers when co-producing ADM with private developers. This is to the cost of responsibility, accountability and transparency when using public ADM.

### A. The Focus on the ‘Final Decision’

The first limitation of the law on non-delegation is its focus on delegation of the ‘final decision’, to the exclusion of considering the decision-making process and exercises of discretion that shape that ‘final decision’.

A decision-maker cannot take the recommendation of another as determinative where the decision-maker cannot lawfully delegate the decision to that other. However, it is permissible to take into account recommendations provided that one does not completely exclude the consideration of other relevant considerations. In *Lavender v Minister of Housing and Local Government*,^48^ the court held that a policy by the Minister of Housing and Local Government (the Minister) to refuse the lease of land for mineral working unless the Minister of Agriculture was not opposed to it was unlawful. This was because, although the Minister was entitled to have a policy and to obtain other ministerial views,^49^ he had ‘in effect inhibited himself from exercising a proper discretion’.^50^ The reality of the policy had eliminated ‘all the material considerations save only the consideration … that the Minister of Agriculture objects’.^51^ There was, therefore, an unlawful fetter on the Minister’s discretion.

However, provided that the final decision is appropriately retained, even great influence from an external recommendation or interim decision is acceptable. This was a consideration in the reasoning of the House of Lords in *R v Secretary of State for the Home Department, Ex parte Oladehinde*.^52^ The case considered whether immigration officials could act on behalf of the Secretary of State in making deportation decisions under section 3(5)(a) of the Immigration Act 1971. Although Lord Griffiths accepted that an initial decision to deport was ‘a serious matter setting in motion the deportation procedure which will gather a momentum that may be difficult to reverse’, he considered it relevant that the ‘initial decision to deport is in a sense provisional as the case is again reviewed before the Secretary of State is invited to sign the deportation order’.^53^ The court therefore concluded that the initial decision was delegable, in the absence of other statutory limitations on delegation and considering the needs of administrative workability, notwithstanding its seriousness and difficulty to reverse.^54^

In *Noon v Matthews*,^55^ the High Court considered whether the Conservators of the River Cam could lawfully delegate the power to institute and bring by-law prosecutions to a river manager.^56^ Beatson LJ explained that ‘where the exercise of the power in question is not final or conclusive … a less strict approach is taken and authority to delegate is likely to be implied’.^57^ The court considered that ‘in determining the extent of implied authority to delegate the fact that the opinion … was not conclusive and that there was an appeal … [was] helpful’.^58^

This is a particularly important limitation in the context of public ADM for two reasons. First, it severely limits the application of the principle of non-delegation to augmented decision-making processes, which make recommendations or initial decisions, which are then finalised by a human decision-maker. As the decision-maker has retained the ‘final’ decision, any delegation is likely to be permissible. Secondly, it severely impacts on the legality of fully automated public decision-making, which produces final outcomes, because all such ‘final’ decisions must be lawfully delegated to it by the decision-maker, irrespective of the degree of input of that decision-maker into the design, development and specifications of the algorithm. The focus on the ‘final’ decision makes the law on non-delegation both under- and over-inclusive when applied to ADM.

This focus on the ‘final’ decision holds, in relation to human decision-making, even where an initial decision that feeds into a ‘final’ decision is both given significant weight and is difficult for the final decision maker to evaluate independently. This can be seen in *R (New College London Ltd) v Secretary of State for the Home Department*,^59^ in which the Supreme Court rejected the argument that a Confirmation of Acceptance for Studies (CAS) from a sponsoring educational institution amounted to an unlawful delegation of the Secretary of State’s power to grant leave to enter or remain. This was because ‘leave to enter or remain continues to be the responsibility of immigration officers and the Secretary of State, who *retain the last word* in each individual case by virtue of the general grounds of refusal’.^60^ As a ‘significant number’ of applicants with a CAS were refused leave to enter or remain, a CAS was ‘not tantamount to leave to enter or remain’ but merely ‘strong but not conclusive evidence of some of the matters which are relevant upon the migrant’s application for leave to enter or remain’.^61^

There is an important parallel between reliance on a CAS as ‘strong but not conclusive evidence’ of relevant matters and reliance on augmented decision-making recommendations, found in decision-support tools. There is undoubtedly a considerable amount of discretion in the acceptance of a student onto a course, based on the evaluation of information about the student and deciding what weight to place on different variables. Similarly, a decision-support tool will determine the weight to be attached to different inputs when applying its algorithmic model to produce a recommendation. Important design choices, which shape those recommendations, involve the exercise of discretion. The ‘final’ decision-maker may lack access to the same information or may lack the time or expertise to scrutinise an interim decision or recommendation, so may treat it as ‘strong but not conclusive evidence’ of relevant matters. Based on the analysis in *New College London Ltd*, there would be little scope to challenge the appropriateness of using such recommendations, provided the ‘final’ decision remains in the decision-maker’s hands.

It is not obvious that the approach taken to human decision-making should be applied to ADM. Even if the law is to accommodate public decision-makers in drawing upon external human expertise and judgement, it does not follow that the same approach should apply to ADM. The latter creates opportunities to consider and make design choices that influence the quality and nature of the recommendation, which might not be as readily available in hierarchical, bureaucratic, human decision-making systems. We should welcome the opportunity to make these processes more transparent, contestable and adaptable than might be possible with human expertise and experience. Applying the same approach to ADM as to human decision-making unnecessarily obscures relevant design choices from determination at a suitable level of seniority, and in doing so misses opportunities for responsibility, accountability and transparency.

An approach that is arguably acceptable, perhaps even necessary, for hierarchical, human decision-making processes may not be so for ADM. For example, in *R (Clarke) v Holliday*,^62^ Murray J considered whether an Inquiry Chair had unlawfully delegated decision-making functions to a member of staff of the Inquiry by ‘permitting them to reach provisional findings at the potential criticism stage’.^63^ He reasoned that it was ‘clear that at the potential criticism stage, no decisions had been made’ and ‘potential criticisms were formulated as a *tool* for exploring the issues with each claimant’.^64^ The inquiry team could compile such criticisms on behalf of the inquiry chair, as ‘sole responsibility for formulating and adopting final decisions as to the criticisms and recommendations … remained throughout’ with the chair.^65^ They were ‘all potential criticisms only and … were formulated as a tool to explore the relevant evidence and issues arising out of the Inquiry’.^66^ While this might be appropriate for a senior chair assisted by staff, aware of the limitations of human information-gathering techniques and supported by a detailed inquiry procedure to put potential criticisms to their subjects and receive further responses and evidence, it is not an approach that translates equally well to augmented decision-making processes, where decision-makers will not necessarily have the experience or knowledge of the limitations of the processes that provide them with recommendations or provisional findings.

### B. An ‘All or Nothing’ Approach

The second limitation of the law on non-delegation is a tendency in the law to view discretionary powers as either wholly delegable or wholly non-delegable. The law does little to consider *how much discretion* over policy and strategy *could* be retained by a named decision-maker before the remainder can be delegated. This limits the ability of the law to isolate the most important issues for personal decision by a decision-maker: delegation is normally understood as ‘all or nothing’.^67^

This tendency is amplified by the importance given to the ‘inevitability’ of delegation when considering whether a power to delegate can be implied. The perceived inevitability of delegation can drive a conclusion that the power to delegate is implied in the relevant power, to make it workable in practice. This feature creates a particularly permissive approach to delegation when combined with the ‘all or nothing’ approach to the permissibility of delegation. It presents a significant limit on all restrictions on delegation, including delegation concerning public ADM, because the proliferation of statutory powers and functions and the scale of public decision-making make the possibility of the retention of discretion in the hands of any one person very unlikely.

This limits the ability of the law to address the retention of discretion in the context of ADM because such technologies are often deployed precisely to manage decision-making with large caseloads or decisions based on large amounts of complex data. It is often precisely because one person cannot personally attend to the task that ADM is attractive, either through full automation or with decision-support tools that augment human processes. If the law on non-delegation considers the inevitability of delegation based on an ‘all or nothing’ approach to delegation, then a permission to delegate will normally be implied. This means that the most important design choices, which are more confined in scope and have the most serious implications for decision-making, are not severed from the part of the decision-making process that must inevitably be delegated to others. It prevents a more nuanced approach to delegable and non-delegable parts of complex decision-making processes. It is important to consider whether an alternative approach, based on isolating the more important parts of a discretionary power, might be better, especially in the context of ADM.

The inevitability of delegation was a key reason given by Lord Greene MR in *Carltona Ltd v Commissioners of Works*, recognising that ‘in the administration of government in this country the functions which are given to ministers (and constitutionally properly given to ministers because they are constitutionally responsible) are functions so multifarious that no minister could ever personally attend to them’.^68^ It was necessary for civil servants to act on behalf of the minister, because otherwise ‘public business could not be carried on’.^69^

The approach of courts to the assessment of ‘inevitability’ has not been entirely consistent. Sometimes the inevitability of delegation is conceived as a general inability to personally exercise all statutory functions, leading to the inevitability of delegation. This is one possible interpretation of Lord Greene’s reasoning in *Carltona*. In *R v Secretary of State for the Home Department, Ex p Doody*, Staughton LJ expressed the point as a general consideration of parliamentary intent that

Parliament must be well aware of the great burden that is imposed on senior ministers, who not only take charge of their departments but also speak for them in Parliament, attend meetings of the Cabinet and its committees, and see to their constituency affairs if they are members of the House of Commons.^70^

There was therefore nothing irrational in devolving a task to a junior minister.^71^ Staughton LJ considered the whole of the minister’s obligations when considering the inevitability of delegation. The rationale was repeated in *R v Secretary of State for the Home Department, Ep p Sherwin* by Kennedy LJ, who argued that it had

long been recognised that in modern government no Secretary of State can personally do everything that is required of him or her, so many acts in fact performed by others are accepted in law as acts of the Secretary of State.^72^

This approach is also apparent in cases involving statutory office holders, rather than ministerial office holders. The High Court considered the whole statutory role of the Metropolitan Police Commissioner in *DPP v Haw*. Lord Phillips CJ argued that ‘where the responsibilities of the office created by statute are such that delegation is inevitable, there will be an implied power to delegate … unless the statute conferring them, expressly or by implication, provides to the contrary’.^73^ This meant that, ‘having regard to the statutory role of the commissioner, one would expect Parliament … to confer them on the commissioner and to leave him to delegate the exercise of those powers as appropriate’.^74^ In light of the ‘practicalities’, Lord Phillips CJ held that ‘it is plain that Parliament cannot have intended that the commissioner should determine the conditions himself’, noting evidence of the high number of applications and the ‘technical’ nature of determining conditions.^75^ In *R (Hamill) v Chelmsford Justices*, Aikens LJ held that it was ‘inevitable that such a duty will be delegated, given the weight of duties and powers that fall on chief constables in practice’, and that ‘nothing in the 2003 Act either expressly or by implication … indicates that the duty … cannot be delegated’.^76^ The focus in these cases was on the practical burden of all of the decision-maker’s duties and powers taken together.

Other cases have focused instead on the inevitability of delegation in the exercise of a particular power. For example, in *Oladehinde*, Lord Griffiths explained that it was

obvious that the Secretary of State cannot personally take every decision to deport an immigrant who is in breach of his condition of entry or is an overstayer. The decision must be taken by a person of suitable seniority in the Home Office for whom the Home Secretary accepts responsibility.^77^

In *Castle v DPP*, Pitchford LJ argued that it was ‘completely unrealistic to think that the Secretary of State could give his personal attention to the traffic speed restriction orders needed to keep traffic on the motorways of England and Wales working as they need’.^78^

Given that the inevitability of delegation in relation to a particular power makes an even stronger case for the permissibility of delegation than inevitability flowing from the general administrative burden on ministers, there is not necessarily any difference in approach, rather than an additional and stronger reason for a conclusion of inevitability. However, the most recent Supreme Court decision on delegation demonstrates a willingness to ask whether delegation is necessary in relation to a particular power, without relying on the general administrative burden on ministers.

In *R v Adams*, the Supreme Court held that there was ‘no evidence that at the time of making the [interim custody order], it would have been unduly onerous for the Secretary of State … personally to consider each application’, and indeed pointed to evidence that a successor Secretary of State in March 1974 being able to consider all such orders personally was ‘a clear indication that it should not have been impossibly difficult … to do the same in July 1973’.^79^ That there was no reason to apprehend that a personal power ‘would place an impossible burden on the Secretary of State’ was a ‘factor that militates towards the conclusion’ that the power was a personal one of the Secretary of State.^80^ This is perhaps some indication that the courts are willing to deny the inevitability of delegation in relation to a particular power. However, the conclusion can only be a tentative one, because the Supreme Court ultimately grounded its reasoning in express statutory indications that the power was a personal power, and therefore could not be delegated, and the power to detain without trial was a particularly serious matter.^81^

The judgment of the High Court in *Noon v Matthews* suggests that an alternative approach is possible, but it is not one found in the reasoning of higher authorities.^82^ It helps to illustrate what is lost by treating delegation as an ‘all or nothing’ matter. In that case, the High Court made a more nuanced distinction between ‘policy’ matters and residual matters for the purposes of lawful delegation. The case concerned the permissibility of delegating decisions relating to the prosecution of by-laws from the Conservators of the River Cam to a river manager. The court held that the Conservators could not delegate the function of bringing by-law prosecutions to a river manager ‘completely’.^83^ It was the *extent* of delegation that was in issue, rather than whether the power *as a whole* could be delegated or not.

This was important because, once the court accepted that it could divide a discretionary power into delegable and non-delegable parts, it was easier for the court to question the *inevitability* of delegation. The court explained that an implied power to delegate was likely ‘where the responsibilities of the person or body named in the statute are such that the court considers delegation is inevitable’,^84^ before distinguishing between the inevitability of delegating some of its functions but not delegating all of its discretion in relation to policy matters:

It does not follow that because it is inevitable that the physical labour needed to survey the river and remove impediments, or to erect toll houses and piers will inevitably be done by others that the Conservators are impliedly authorised to delegate entirely decisions as to when and where to survey or to place a pier or a toll house.^85^

The shift to a more granular approach, rather than an ‘all or nothing’ approach, to delegation therefore mitigated the tendency of ‘inevitability’ to drive the conclusion that a power must be delegable. However, once it is accepted that only a *partial* delegation of discretionary power might be lawful, it becomes necessary to determine which parts of that power are delegable and which are not. Although this has the benefit of greater granularity, mitigates the inevitability of delegation and therefore allows more scope to selectively retain discretion, responsibility and accountability, it requires courts to make a justifiable division between those delegable and non-delegable parts.

In *Noon v Matthews*, the court argued that ‘a distinction must be made between the determination of policy on such matters and the operational execution of such policy’.^86^ The court was not unaware of the ‘difficulties at the margin of locating the boundaries of those categories’, but proceeded on that basis nonetheless.^87^ This meant that while the Conservators were ‘not impliedly authorised to delegate broad policy’, they could ‘delegate the implementation of such policies to officers who will have some discretion as to how, operationally, to execute the policy in question’.^88^

The court rejected an alternative submission that the appropriate division was one in which the Conservators could lawfully delegate to the manager the preparation and processing of a prosecution while retaining discretion over the final review and approval of that prosecution.^89^ That is important, because it demonstrates that the court was willing to countenance the delegation of ‘final’ decisions, guided by a policy set by the Conservators, rather than insisting on the retention of all ‘final’ decisions. This has important implications for delegation in the context of public ADM because fully automated decision-making, which produces ‘final’ decisions, might not be excluded by the retention of some decision-making discretion over the ‘policy’ it reflects if that ‘policy’ relates to important design parameters of the algorithm. Abandoning an ‘all or nothing’ approach to delegation can therefore also mitigate the limitations that flow from a focus on the ‘final’ decision.

The court considered the seriousness of a decision to prosecute against the inevitably of ‘some delegation to the most senior officer’^90^ before determining that while ‘it is for the Conservators to set the general policy regarding prosecutions … as far as individual prosecutions within such general policy are concerned, there is power in their senior officer … to make the operational decisions’.^91^ As the Conservators ‘had decided on a policy of prosecuting unlawful punt operators’, it was lawful to delegate to the river manager individual decisions to prosecute particular cases in accordance with that policy.^92^ It should be noted that the alternative submission for a distinction between the preparation of a prosecution and the final decision to prosecute was similarly grounded in arguments about the seriousness of the decision and the fact that it was not inevitable that the Conservators would need to delegate some decisions to prosecute.^93^ They were of a volume that could be decided individually by the Conservators. This indicates some uncertainty about the role of seriousness and inevitability in drawing the distinction between delegable and non-delegable portions of a discretionary power.

The terminology of the policy/operational distinction should also be treated with caution because there is no simple analytical line between policy and operation in a decision-making process, as the court noted. Choices which require trade-offs may often be made in order to operationalise a policy, depending on the determinacy of the policy, which could themselves be characterised as ‘policy’ choices. Some policies may be intentionally abstract, setting targets and objectives for a delegate to pursue according to the means they think fit. Some policies are more detailed but nevertheless raise questions about their proper application or require decisions, even if provisional, about how to operationalise them.

There is a risk that a policy/operational distinction becomes a mere conclusory label for a non-delegable/delegable distinction justified by reference to other considerations. This is, in reality, the approach adopted in *Noon v Matthews*, balancing considerations of seriousness and administrative inevitability, notwithstanding the language of policy and operational decisions. It is an approach that could be adopted more widely in relation to the application of the *Carltona* principle and non-delegation. The claim here is not that it is a watertight analytic distinction, but that it might be preferable to an ‘all or nothing’ approach that too easily concludes that important policy decisions must inevitability be delegable with the remainder of a discretionary power.

A potential obstacle to this approach lies in the reluctance of the courts to attach significant weight to the seriousness of the consequences of a decision when considering the permissibility of delegation. A concept of seriousness, importance or gravity would need to sit behind the labels of a policy/operational distinction and would requires difficult judgments in marginal cases. This is not an insurmountable problem, as deference to the decision-maker seeking to delegate could be exercised at those margins. The idea of seriousness is not, in principle, more obscure than other concepts in administrative law, and the importance of a decision is a factor taken into account in reasoning in relation to many grounds of review, such as procedural fairness.^94^

However, although the Supreme Court in *Adams* recently recognised that ‘the seriousness of the consequences is a consideration to be taken into account in deciding whether a power must be exercised … personally’,^95^ the wider case law on the *Carltona* principle and non-delegation demonstrates a reluctance to attach significant weight to this consideration.

Even in *Adams* itself, the fact that the power was a ‘momentous one’, a power to indefinitely detain without trial, only served to reinforce the court’s conclusion based on statutory construction, because it provided ‘an insight into Parliament’s intention’.^96^ The seriousness of the decision was therefore *merely supplemental* to other statutory express or necessarily implied indications that a power must be personally exercised. The decisive factor was the statutory language, which distinguished between the power of the ‘Secretary of State [to] make an order’ and the requirement for an ‘interim custody order of the Secretary of State’ to be ‘signed by a Secretary of State, Minister of State or Under-Secretary of State’.^97^ There was, according to the Supreme Court, a ‘distinct segregation of roles’ with ‘quite different treatment’,^98^ which could not have been other than ‘deliberate’.^99^ It therefore must denote a personal power.^100^

The Supreme Court in *Adams also* considered *R v Harper* correctly decided,^101^ despite its reliance on Brightman J’s reasoning regarding the seriousness of the subject matter of a decision in *In re Golden Chemicals Products Ltd*,^102^ which the Supreme Court in *Adams* rejected.^103^ Brightman J had rejected a distinction between powers that must be exercised personally and those that can be exercised by departmental officials, based on the importance of the decision.^104^ He explained that while importance might create a ‘political necessity’ for personal attention, it did not create a ‘legal necessity’.^105^ The Supreme Court in *Adams* considered that Brightman J had held that ‘the seriousness of the subject matter’ was ‘not a consideration which was relevant at all’, and rejected that claim.^106^ However, in relation to *Harper*, the decision remained correctly decided because there was ‘nothing in the legislation which gave rise to the possibility of implying any restriction’ on delegation, nor was there ‘express restriction’.^107^ This was despite the fact that *Harper* concerned detention beyond 48 hours under the Prevention of Terrorism (Temporary Provisions) Act 1984, which implies that even serious consequences for individual liberty may not be sufficient in the absence of *other* express or implied statutory restrictions on the power to devolve or delegate. The Supreme Court in *Adams* regarded it as a ‘straightforward application’ of *Carltona*.^108^

Similarly, in *re McCafferty’s Application*,^109^ the Court of Appeal in Northern Ireland, despite accepting that the ‘importance of the subject matter’ was one of the ‘implied factors to be considered’,^110^ held that a revocation of a release on licence by a junior minister was lawful.^111^ This was because there was ‘nothing in either the framework or the language of the 1995 Act that indicates a contrary Parliamentary intention’.^112^

However, if this is so, the Supreme Court in *Adams* and the Court of Appeal in Northern Ireland in *McCafferty* regarded seriousness as a merely supplementary consideration in combination with other aspects of statutory interpretation. This would present a challenge for using seriousness to ground a policy/operational distinction, because the relevant statutory power will not normally contain implicit or explicit indications of how that distinction is to be drawn.

The Supreme Court in *Adams* also did not question the correctness of the judgment of the Court of Appeal in *R v Skinner*.^113^ In *Skinner*, the Court of Appeal held that the Secretary of State was not required to personally approve alcohol breath tests required by section 7(1) of the Road Safety Act 1967.^114^ As Brightman J had observed in *Golden Chemicals*, the accuracy of breath test equipment was of ‘vital importance to every motorist’, with implications for the arrest of innocent motorists or release of intoxicated ones.^115^ The Court of Appeal in *Skinner* rejected a submission seeking to distinguish *Carltona* on the basis that the decision was an ‘isolated and vitally important matter [that] might well have occupied the Minister’s personal attention’ because there was ‘in principle no obligation upon the Minister to give it his personal attention’.^116^ The Supreme Court in *Adams* left this untouched, and the problems that *Skinner* presents for an alternative approach that seeks the retention of important design choices for ADM at a sufficiently high level of seniority are clear. If ensuring that accuracy of breath tests is a duty that need not be personally retained, then there are challenges for requiring important design choices in public ADM to be personally retained.

In *Golden Chemicals*, Brightman J had questioned how a distinction based on the importance of the matter was to be drawn: ‘A distinction between a case which involves a serious invasion of the freedom or property rights of the subject, and a case which involves a similar invasion that is not serious, seems to me to be impossibly vague.’^117^

The problem is not a minor one if seriousness is to be a *determining factor*, because seriousness is a matter of degree and a non-arbitrary threshold would need to be identified. Determining seriousness against a threshold also requires an evaluation of the relative importance of various rights and interests. It is also not clear whether ‘the seriousness of the consequences’ refers only to decisions that are individually important, such as the loss of liberty without trial involved in the interim custody order in *Adams*, or could further extend to matters of marginal consequence in individual decisions whose cumulative effects can be described as serious. The Supreme Court in *Adams* only referred to the consideration as the ‘gravity of the consequences flowing from the exercise of the power’.^118^ Although this is understandable on the facts of *Adams*, which concerned the delegation of an order in relation to the detention of Gerry Adams, it leaves unresolved the proper approach to seriousness where ADM perpetuates bias over larger populations. Although in isolation such bias might have a marginal impact in each case, its culminative effects can work serious injustices over populations.

Another indicator that the courts would be reluctant to give the seriousness of a decision decisive weight in reasoning about the permissibility of non-delegation can be found in their presumptions about statutory intent in relation to *Carltona*. Although the Supreme Court in *Adams* did not come to a ‘firm conclusion’ about the status of the *Carltona* principle as a statutory presumption^119^ because it would in any event be displaced by clear statutory language in that case,^120^ and it denied that there was ‘a presumption in law that the principle must be taken to apply unless it has been removed by express statutory language’,^121^ it did note that Parliament legislates against the background of the *Carltona* principle.^122^ It was therefore relevant that Parliament had explicitly displaced the *Carltona* principle on occasion, and therefore, where it had not done so, this was a relevant consideration in construing a statutory power as delegable.^123^

It might therefore be difficult to persuade the courts to take a more active role in distinguishing between policy and operational parts of a discretionary power, and requiring the retention of the important policy choices in the development of public ADM. The reluctance to use the seriousness of a decision as determinative, the focus on statutory language and *Carltona* as a well-established part of the legislative background, and reliance on the inevitability of delegation all combine with the ‘all or nothing’ approach of the courts to present real challenges to a more nuanced approach to the retention of important aspects of discretionary decision-making.

### C. The Scope of the Carltona Principle

The third limitation of the law on non-delegation lies in the scope of the *Carltona* principle itself. Its scope is both under- and over-inclusive because it is very permissive of delegation internally of government and very restrictive of delegation externally to government, which presents problems where public ADM is co-produced between public authorities and private developers. Although there is little evidence of the development of public ADM entirely internally to government, the permissive approach within government would leave little scope to require the retention of important design choices by senior officials. In relation to the co-production of ADM by officials and private developers, there is serious doubt as to whether decisions can be delegated to private contractors under the *Carltona* principle. This has the potential to inhibit the adoption of fully automated decision-making where a private developer provides the technology. Alternatively, it has the potential to encourage greater reliance on a ‘final decision’ being taken within government, using decision-support tools to augment a human decision. However, oversight of the ‘final decision’ provides limited reassurance that important design choices and the limitations of the algorithm will be properly understood and reflected by those decision-makers.

The *Carltona* principle, as traditionally conceived, was limited to departmental officials. Lord Greene MR, in the judgment of the Court of Appeal in *Carltona*, held that the ‘duties imposed upon ministers and the powers given to ministers’ could normally be ‘exercised under the authority of the ministers by responsible officials of the department’.^124^ A requisition order by an assistant secretary in the Ministry of Works and Planning on behalf of the Commissioners of Works was therefore lawfully made on behalf of the Secretary of State.

Later, it was questioned whether the *Carltona* principle could extend to civil servants in executive agencies of government departments.^125^ In *Sherwin*,^126^ the High Court rejected the contention that the creation of a ‘Next Steps’ agency altered the relationship between minister and agency worker ‘so fundamentally that the *Carltona* principle could not operate’.^127^ Such an agency carried out ‘executive functions of government within a policy and resources framework set by a department’, separating ‘main strategic control’ and ‘policy objective and budgets’ from independent management to ‘decide how those objectives are met’.^128^ Although Next Steps agencies relied on a policy/operational distinction, that distinction had already been made within departments before their creation.^129^ The Framework Document for the Benefits Agency provided that it ‘acts on behalf of and in accordance with any directions, where appropriate, of the Secretary of State’ and ‘Ministers remain accountable to Parliament for the full range of their responsibilities’, although they ‘do not normally interfere in the day to day running of the Agency’.^130^ Freedland describes this as a form of quasi-contractual relationship.^131^ The court therefore held that the creation had ‘no effect whatsoever on the operation of the *Carltona* principle’:^132^ the relevant decision had been ‘taken by a person of suitable seniority … for whom the Secretary of State accepts responsibility’.^133^

Similarly, in *Castle*, the High Court considered whether an employee of the Highways Agency could act on behalf of the Secretary of State for Transport to make traffic restriction orders pursuant to the Road Traffic Regulation Act 1984.^134^ Pitchford LJ observed that

if the observations of Lord Greene MR as to the realities of departmental government were as he described them in 1943, then how much more apposite must they be to modern conditions in which administration decisions and secondary legislation occur every second of the working day.^135^

Explaining that it was ‘constitutional responsibility’ that enabled the court in *Sherwin* to apply *Carltona* to a Next Steps agency, Pitchford LJ held that the Highways Agency had an ‘equivalent framework document’ which required the Highways Agency to ‘deliver the policy of the department’ regarding certain operations and made clear that ‘the Secretary of State is accountable to Parliament for the agency’.^136^ It was therefore in law the ‘alter ego of the Department for Transport’ in those areas.^137^ Provided that the decision was devolved to ‘those properly qualified to make the judgment’, the devolution was lawful.^138^

The *Carltona* principle cannot be used to devolve or delegate decision-making from one minister to another^139^ or from a minister to an independent statutory office holder.^140^ In *Bourgass*, the Supreme Court considered whether it was lawful for the Secretary of State to delegate his power to authorise prisoner segregation beyond 72 hours to prison governors or officers. Lord Reed reasoned that a departmental official was ‘in a different constitutional position’ from a statutory office holder.^141^ This was because while the minister was ‘constitutionally responsible’ for a departmental official, independent statutory office holders were ‘constitutionally responsible’ themselves: it was the ‘constitutional separation’ between the Secretary of State and the independent statutory officer that ‘prevented a lawful devolution of [the] power’ from one to the other.^142^

Additionally, Lord Reed emphasised that the separation of responsibilities between the governor and the Secretary of State in authorising the segregation of prisoners was a ‘safeguard for the prisoner’.^143^ Permitting the decision to be taken by a governor or prison officer would defeat the purpose of the rule.^144^ Similarly, in *Oladehinde*, although the House of Lords held it was lawful for immigration officials to act on behalf of the Secretary of State in making deportation decisions under section 3(5)(a) of the Immigration Act 1971,^145^ it could not be devolved to an official where the devolution would ‘conflict with or embarrass them in the discharge of their specific statutory duties’.^146^ Nor can delegation take place where it would negate the personal qualifications of the named decision-maker. In *R (Chief Constable of the West Midlands Police) v Birmingham Justices*,^147^ the High Court distinguished between powers conferred on an office holder at the ‘apex of an organisation itself composed of other holders or otherwise hierarchically structured’ and powers conferred ‘because of the personal qualifications of the individual holder’, such as a ‘medical officer of health’ or a ‘statutory inspector’.^148^ This produces some constraints on the use of public ADM where the identity of the decision-maker is itself a safeguard within the statutory scheme or reflects personal qualifications and expertise. Public ADM cannot circumvent such safeguards and qualifications.

In addition to delegation to executive agencies established by a parent department as part of its organisational structure, similar principles have applied to the scope of implied delegation by statutory office holders within their organisations.^149^ The law on non-delegation is therefore very permissive of delegation within government or public authority structures, so long as accountability for decisions nominally rests with the delegator.

This permissive approach is further reflected in the level of scrutiny applied to a decision to delegate to a particular official. In *Chief Constable of the West Midlands Police*,^150^ the court distinguished between identifying whether someone was ‘*a* proper and appropriate agent’ and holding that they were ‘*the* proper and appropriate agent’.^151^ To do the latter would be ‘not to interpret but to legislate’: ‘the question for a court is not how it considers that [one] should be exercising [a] power of delegation; it is whether [one] has exercised it within permissible limits’.^152^ The court noted that it was for the delegator ‘to decide who is best suited’ and it was not for the court to ‘second-guess’ that decision unless it was ‘irrational or otherwise beyond [their] powers’.^153^ The decision as to who is ‘suitable [was] primarily for the office-holder to decide’.^154^ The Court of Appeal in *Doody* also applied an irrationality analysis to a decision to devolve a decision to a junior minister.^155^ This means that despite there being considerable case law that makes reference to delegation that it is ‘suitable to their grading and experience’,^156^ to someone ‘of suitable seniority’,^157^ one ‘properly qualified to make the judgment’,^158^ ‘suitable’^159^ or of ‘seniority … of an appropriate level having regard to the nature of the duty itself’,^160^ these standards are undercut by mere rationality review of a decision to delegate to a particular individual.

The importance of this is that once it has been determined that a power is delegable, there is very little scope to scrutinise the suitability of the delegate outside irrational choices. In combination with the ‘all or nothing’ approach, there is little to prevent ADM design choices of the greatest gravity from being delegated within government. By contrast, it is not possible to delegate a power beyond departmental or agency officials or the employees of a statutory office holder. In relation to some ministerial functions, that traditional understanding has resulted in the creation of cumbersome legislative provisions, such as those found in the Deregulation and Contracting Out Act 1994. However, given the focus of non-delegation on the ‘final decision’, this is potentially easy to circumvent when using ADM by relying on an official within the structures for which the ultimate decision-maker is nominally responsible to review an algorithmic recommendation or initial decision and decide to endorse it. In reality, of course, there might be little to reassure that such a ‘final decision’ is not merely a rubber stamp, unduly deferential, made without full understanding of the limitations of the decision-support tool or subject to automation bias. The permissive focus on that ‘final decision’, taken by a departmental official whose suitability merely cannot be said to be irrational, and legitimated by the administrative ‘inevitability’ of delegation against a counterfactual of personal retention of the entirely of ‘all’ decisions under that power by one named decision-maker, does much to obscure the important points of decision in the design of the tool itself.

## 4. The Scale of the Challenge for Non-delegation and Public ADM

As it currently stands, the principle of non-delegation has significant limitations in its ability to ensure that important design choices are appropriately retained by accountable and responsible public decision-makers, so that those choices are transparent and contestable. It could only be adapted to the modern machinery of government if these significant limitations are overcome. Reliance on such development by the courts might not be readily forthcoming. It would require a willingness on the part of the courts to recognise that the ‘final’ decision is not necessarily the most important, nor a sufficient safeguard to oversee delegated provisional decisions. It would require the courts to move away from an ‘all or nothing’ approach to the permissibility of delegation, to distinguish between delegable and non-delegable aspects of discretionary decision-making.

Without an ‘all or nothing’ approach, inevitability would need to be considered in a more granular way and considerations of seriousness given a greater role in the permissibility of delegation. The *Carltona* principle and similar principles in the wider law on delegation would need to adapt, along with assumptions about the legislative intent that can safely be assumed from an absence of explicit restrictions on delegation. The permissive approach to delegation within ministerial departments and public authorities would need to be curtailed, and there would need to be a greater openness to delegation to those outside those structures, provided that adequate frameworks were maintained to ensure responsibility, accountability and transparency. This is undoubtedly a tall order for judicial development alone. There is a clear risk that the courts will be slow to, or fail to, adapt to the changing machinery of government. It would no doubt be challenged as too innovative a task for the courts, where Parliament has long legislated against a relatively stable body of legal doctrine on the permissibility of delegation. It would require the right case to present itself. This would itself be a challenge with the odds stacked against success, given the current approach of the courts.

While it is not inevitable that new machineries of government will undermine responsibility, accountability and transparency, new ways of thinking about how responsibility, accountability and transparency can be appropriately retained in the design, development and deployment of those technologies will be required as their use expands in public decision-making.

## Footnotes

2 See generally Roger Masterman and Colin Murray, *Constitutional and Administrative Law* (CUP 2022) 333–5 and 630–2; William Wade, Christopher Forsyth and Julian Ghosh, *Administrative Law* (OUP 2022) 262.

3
*Carltona v Commissioners for Works* [1943] 2 All ER 560 (CA).

4
*R (Bourgass) v Secretary of State for Justice* [2015] UKSC 54, [2016] AC 384.

5 See *R v Secretary of State for the Home Department, Ex p Sherwin* (1996) 32 BMLR 1 (QB); *Castle v DPP* [2014] EWHC 587 (Admin), [2014] 1 WLR 4279.

6 See *R (Chief Constable of the West Midlands Police) v Birmingham Justices* [2002] EWHC 1087 (Admin), [2003] Crim LR 37 [9]–[12]; *DPP v Haw* [2007] EWHC 1931 (Admin), [2008] 1 WLR 379 [33]; *R (Hamill) v Chelmsford Justices* [2014] EWHC 2799 (Admin), [2015] 1 WLR 1798 [64].

7 See Masterman and Murray (n 1) 333–5 and 630–2; Wade, Forsyth and Ghosh (n 1) 262.

8
*Lewisham Borough Council v Roberts* [1949] 2 KB 608, 621, [1949] 1 All ER 815.

9 See generally Paul Craig, *Administrative Law* (Sweet & Maxwell 2021) ch 10.

10 Public Law Project, ‘The Tracking Automated Government Register’ (9 February 2023) <https://publiclawproject.org.uk/resources/the-tracking-automated-government-register/> accessed 21 November 2024.

11 UK Government, ‘Algorithmic Transparency Recording Hub’ (5 January 2023) <www.gov.uk/government/collections/algorithmic-transparency-recording-standard-hub> accessed 21 November 2024.

12 See Albert Sanchez-Graells, ‘Responsibly Buying Artificial Intelligence: A “Regulatory Hallucination”’ [2024] CLP 1.

13 See Jennifer Cobbe, Michael Veale and Jatinder Singh, ‘Understanding Accountability in Algorithmic Supply Chains’ in *FAccT’23: Proceedings of the 2023 ACM Conference on Fairness, Accountability, and Transparency* 1186 <https://doi.org/10.1145/3593013.3594073> accessed 7 February 2025.

14 See eg David Leslie, *Understanding Artificial Intelligence Ethics and Safety: A Guide for the Responsible Design and Implementation of AI Systems in the Public Sector* (The Alan Turing Institute 2019) <www.turing.ac.uk/sites/default/files/2019-06/understanding_artificial_intelligence_ethics_and_safety.pdf> accessed 7 February 2025.

15 Karen Yeung and Martin Lodge, ‘Algorithmic Regulation: An Introduction’ in Karen Yeung and Martin Lodge (eds), *Algorithmic Regulation* (OUP 2019) 5.

16 eg Regulation (EU) 2016/679 of the European Parliament and of 27 April 2016 on the protection of natural persons with regard to the processing of personal data and on the free movement of such data, repealing Directive 95/46/EC (General Data Protection Regulation) [2016] OJ L119/01, art 22, which applies only to ‘solely’ automated individual decision-making.

17 Ed Main and Richard Watson, ‘The English Test that Ruined Thousands of Lives (9 February 2022) <www.bbc.co.uk/news/uk-60264106> accessed 21 November 2024.

18 National Audit Office, ‘Investigation into the Response to Cheating in English Language Tests’ (24 May 2019) <www.nao.org.uk/wp-content/uploads/2019/05/Investigation-into-the-response-to-cheating-in-English-language-tests.pdf> accessed 21 November 2024.

19 See ICO, ‘Enforcement Notice’ (13 November 2018) <www.met.police.uk/SysSiteAssets/media/downloads/force-content/met/about-us/gangs-violence-matrix/ico-enforcement-notice.pdf> accessed 21 November 2024.

20 Metropolitan Police, ‘Gangs Violence Matrix’ (October 2022) <www.met.police.uk/police-forces/metropolitan-police/areas/about-us/about-the-met/gangs-violence-matrix/> accessed 21 November 2024.

21 Amnesty International, ‘What Is the Gangs Matrix?’ (18 May 2020) <www.amnesty.org.uk/london-trident-gangs-matrix-metropolitan-police?utm_source=google&utm_medium=grant&utm_campaign=BRD_AWA_GEN_dynamic-search-ads&utm_content=&gad_source=1&gclid=CjwKCAiArfauBhApEiwAeoB7qGWXrYpxBABeGmrbFzljGas7aclK4aeh64MqC6isBjMhCuTCIp1sExoC8RcQAvD_BwE> accessed 21 November 2024.

22 BBC, ‘A-Levels and GCSEs: How did the Exam Algorithm Work?’ (20 August 2020) <www.bbc.co.uk/news/explainers-53807730> accessed 21 November 2024.

23 Sandra Wachter, Brent Mittlestadt and Luciano Floridi, ‘Why a Right to Explanation of Automated Decision-Making Does Not Exist in the General Data Protection Regulation’ (2017) 7(2) IDPL 76; Sandra Wachter and Brent Mittelstadt, ‘A Right to Reasonable Inferences: Re-thinking Data Protection Law in the Age of Big Data and AI’ [2018] Colum Bus L Rev 443; Sandra Wachter, Brent Mittlestadt and Chris Russell, ‘Counterfactual Explanations Without Opening the Black Box: Automated Decisions and the GDPR’ (2018) 31(2) Harv JL & Tech 841.

24 See Shoshana Zuboff, *The Age of Surveillance Capitalism* (Profile Books 2019); Carissa Véliz, *Privacy Is Power: Why and How You Should Take Back Control of Your Data* (Bantam Press 2020).

25 See Michael Veale and Reuben Binns, ‘Fairer Machine Learning in the Real World: Mitigating Discrimination without Collecting Sensitive Data’ [2017] Big Data & Society 1; Virginia Eubanks, *Automating Inequality: How High-Tech Tools Profile, Police, and Punish the Poor* (St Martin’s Press 2017); Safia Noble, *Algorithms of Oppression: How Search Engines Reinforce Racism* (New York University Press 2018); Ruha Benjamin, *Race after Technology* (Polity Press 2019); Caroline Criado Perez, *Invisible Women: Exposing Data Bias in a World Designed for Men* (Vintage 2019); Sandra Wachter, Brent Mittelstadt and Chris Russell, ‘Why Fairness Cannot Be Automated: Bridging the Gap Between EU Non-Discrimination Law and AI’ (2021) 41(July) CLS Rev <https://papers.ssrn.com/sol3/papers.cfm?abstract_id=3547922> accessed 21 November 2024.

26 See Jeremias Adams-Prassl, ‘What if Your Boss Was an Algorithm? The Rise of Artificial Intelligence at Work’ (2019) 41 Comparative Labor Law & Policy Journal 123.

27 White & Case, ‘AI Watch: Global Regulatory Tracker- European Union’ (18 October 2024) <www.whitecase.com/insight-our-thinking/ai-watch-global-regulatory-tracker-european-union#:~:text=Status%20of%20the%20AI%20Regulations,in%20the%20EU’s%20Official%20Journal> accessed 21 November 2024.

28 UK Government, ‘Algorithmic Transparency’ (n 10).

29 UK Government, ‘Guidelines for AI Procurement’ (8 June 2020) <www.gov.uk/government/publications/guidelines-for-ai-procurement> accessed 21 November.

30 Sanchez-Graells (n 11).

31 See Danielle Keats Citron and Frank Pasquale, ‘The Scored Society: Due Process for Automated Predictions’ (2014) 89 Wash L Rev 1, 10–11; Cary Coglianese and David Lehr, ‘Regulating by Robot: Administrative Decision Making in the Machine-Learning Era’ (2017) 105 Geo LJ 1147; Marion Oswald, ‘Algorithm-Assisted Decision-Making in the Public Sector: Framing the Issues using Administrative Law Rules Governing Discretionary Power’ [2018] Philosophical Transactions A 376; Jennifer Cobbe, ‘Administrative Law and the Machines of Government: Judicial Review of Automated Public-Sector Decision-Making’ (2019) 39(4) LS 636.

32 Cobbe (n 31).

33 Elena Casale, ‘Around the Black Box: Applying the Carltona Principle to Challenge Machine Learning Algorithms in Public Sector Decision-Making’ [2021] LSE Law Review 369. See also broader concerns about the accountability for exercises of administrative discretion through algorithms in Madalina Busuioc, ‘Accountability in the Age of Artificial Intelligence’ in Matthew Flinders and Chris Monaghan (eds), *Questions of Accountability: Prerogatives, Power and Politics* (Hart Publishing 2023); concerning international law in Fleur Johns, ‘Data, Detection, and the Redistribution of the Sensible in International Law’ (2017) 111 AJIL 57.

34 Lord Philip Sales, ‘Algorithms, Artificial Intelligence and the Law’ [2020] JR 46, 10.

35 Lord Philip Sales, ‘Information Law and Automated Governance’ [2023] JR 280, 292.

36 Lord Philip Sales, ‘Judicial Review Methodology in the Automated State’ (presentation for the Conference on Automation in Public Governance—Theory, Practice and Problems, Prato, Italy, September 2024) 8 <https://supremecourt.uk/uploads/speech_lord_sales_2409_157955367d.pdf> accessed 7 February 2025.

37 See Citron and Pasquale (n 30); Finale Doshi-Velez and others, ‘Accountability of AI under the Law: The Role of Explanation’ (Berkman Center Research 2017) <https://papers.ssrn.com/sol3/papers.cfm?abstract_id=3064761> accessed 21 November 2024; Katherine Strandburg, ‘Rulemaking and Inscrutable Automated Decision Tools’ (2019) 119(7) Colum L Rev 1851.

38 Teresa Scantamburlo, Andrew Charlesworth and Nello Cristianini, ‘Machine Decisions and Human Consequences’ in Yeung and Lodge, *Algorithmic Regulation* (n 14) 53–4.

39 Natalia Criado and Jose M Such, ‘Digital Discrimination’ in Yeung and Lodge, *Algorithmic Regulation* (n 14) 89.

40 Karen Yeung, ‘Towards an Understanding of Regulation by Design’ in R Brownsword and K Yeung (eds), *Regulating Technologies: Legal Futures, Regulatory Frames and Technological Fixes* (Hart Publishing 2008); Karen Yeung, ‘Design for Regulation’ in J van den Hoven, PE Vermaas and I van de Poel (eds), *Handbook of Ethics, Values and Technological Design* (Springer 2015); Yeung and Lodge, ‘Algorithmic Regulation: An Introduction’ (n 14).

41 Adam Harkens, ‘Fairness in Algorithmic Decision-Making’ (2019) 1(1) Amicus Curiae 84, 94.

42 Oswald (n 30) 392.

43 Lina Dencik and others, ‘Data Scores as Governance: Investigating Uses of Citizen Scoring in Public Services: Project Report’ (Open Society Foundations 2018) 7.

44 S Jasanoff, *The Ethics of Invention* (WW Norton & Co 2016); Karen Yeung, ‘Why Worry about Decision-Making by Machine?’ in Yeung and Lodge, *Algorithmic Regulation* (n 14) 26.

45 Michael Veale and Irina Brass, ‘Administration by Algorithm? Public Management Meets Public Sector Machine Learning’ in Yeung and Lodge, *Algorithmic Regulation* (n 14) 133.

46 ibid.

47 See eg Stephen P Turner (ed), *The Politics of Expertise* (Routledge 2015); Alfred Moore, *Critical Elitism: Deliberation, Democracy, and the Problem of Expertise* (CUP 2017).

48 (1970) 1 WLR 1231 (QB).

49 ibid 1238

50 ibid 1240

51 ibid 1241.

52 [1991] 1 AC 254 (HL).

53 ibid 301.

54 ibid 300–3.

55 [2014] EWHC 4330 (Admin), [2015] ACD 53.

56 ibid [1].

57 ibid [26].

58 ibid [29].

59 [2013] UKSC 51, [2013] 1 WLR 2358.

60 ibid [19] (emphasis added).

61 ibid [19].

62 [2019] EWHC 3596 (Admin), [2020] ACD 33.

63 ibid [35].

64 ibid [53] (emphasis added).

65 ibid [53] (emphasis added).

66 ibid [56].

67 See eg *Bourgass* (n 3); *Haw* (n 5); *Hamill* (n 5).

68
*Carltona* (n 2) 563.

69 ibid 563.

70 [1993] QB 157 (CA) 196.

71 ibid.

72
*Sherwin* (n 4) [4].

73
*Haw* (n 5) [33].

74 ibid [34].

75 ibid [36].

76 ibid [64].

77
*Oladehinde* (n 51) 300.

78
*Castle* (n 4) [28].

79 [2020] UKSC 19, [2020] 1 WLR 2077 [19].

80 ibid [39].

81 ibid [28]–[37].

82
*Noon v Matthews* (n 54).

83 ibid [3]–[4].

84 ibid.

85 ibid [37].

86 ibid [39].

87 ibid [39].

88 ibid [39].

89 ibid [22].

90 ibid [40].

91 ibid [43].

92 ibid [47].

93 ibid [22].

94 See eg *Doody* (n 69).

95
*Adams* (n 78).

96 ibid [38].

97 ibid [28]–[30].

98 ibid [31].

99 ibid [37].

100 ibid [32].

101 [1990] NI 28 (CA).

102 [1976] Ch 300 (Ch).

103
*Adams* (n 78) [13].

104
*Golden Chemicals* (n 101) 310.

105 ibid 310.

106
*Adams* (n 78) [13].

107 ibid [23].

108 ibid [23].

109 [2009] NICA 59.

110 ibid [17].

111 The relevant power was the Northern Ireland (Remission of Sentences) Act 1995, s 1(3).

112
*McCafferty* (n 108) [17].

113 [1968] 2 QB 700, [1968] 3 WLR 408.

114 ibid.

115
*Golden Chemicals* (n 101) 310.

116
*Skinner* (n 112) 709.

117
*Golden Chemicals* (n 101) 310.

118
*Adams* (n 78) [26].

119 ibid [25].

120 ibid [26].

121 ibid [25].

122 ibid [25].

123 ibid [25].

124
*Carltona* (n 2) 563. See also *Oladehinde* (n 51) 300–3.

125 Mark Freedland, ‘The Rule against Delegation and the *Carltona* Doctrine in an Agency Context’ [1996] PL 19.

126
*Sherwin* (n 4).

127 ibid [5].

128 ibid [6].

129 ibid [8].

130 ibid [7].

131 Freedland (n 124) 20.

132
*Sherwin* (n 4) [9].

133 ibid.

134
*Castle* (n 4).

135 ibid [17].

136 ibid [23].

137 ibid [24].

138 ibid [28].

139
*Lavende* (n 47).

140
*Bourgass* (n 3).

141
*Bourgass* (n 3) [50].

142 ibid [50] and [64].

143 ibid [72].

144 ibid [89].

145
*Oladehinde* (n 51).

146 ibid 303.

147
*Chief Constable of the West Midlands Police* (n 5).

148 ibid [14]. See also *Noon v Matthews* (n 54) [26].

149 See *Chief Constable of the West Midlands Police* (n 5) [9]–[12]; *Haw* (n 5) [33]; *Hamill* (n 5) [64].

150
*Chief Constable of the West Midlands Police* (n 5).

151 ibid [16] (emphasis in original).

152 ibid [16].

153 ibid [16].

154 ibid [19].

155
*Doody* (n 69). The point was subsequently endorsed by the House of Lords [1994] 1 AC 531 (HL).

156
*Oladehinde* (n 51) 303.

157
*Sherwin* (n 4) [9].

158
*Castle* (n 4) [28].

159
*Chief Constable of the West Midlands Police* (n 5) [9]–[10].

160
*Hamill* (n 5) [64]. Lord Phillips CJ used a virtually identical phrase in dicta comments *Haw* (n 5) [29].

